# Supplementary material for: A physiological and histological atlas of reproduction in the North American deer mouse (Peromyscus maniculatus)
Source: PLoS One. 2025 Jun 5;20(6):e0323266. doi: 10.1371/journal.pone.0323266 (PMC12140262; doi:10.1371/journal.pone.0323266)
Supplement: S1 Table — (DOCX) [file pone.0323266.s001.docx]

| **Gestational Stage** | | Dam (N) | Total Implantation Sites Collected (N) | Cryosectioned Implantation Sites (N) |
| --- | --- | --- | --- | --- |
| **Embryonic Day** | **Theiler Stage** |  |  |  |
| e11.5 | TS 14 -15 | 1 | 7 | 2 |
| e12.5 | TS 16 -17 | 2 | 13 | 5 |
| e13.5 | TS 18-19 | 2 | 15 | 5 |
| e14.5 | TS 20 | 1 | 5 | 4 |
| e 15.5 | TS 21 | 1 | 6 | 4 |
| e16.5 | TS 22 | 2 | 13 | 6 |
| e17.5 | TS 22.5 | 1 | 7 | 2 |
| e18.5 | TS 23 | 1 | 8 | 2 |
| e20.5 | TS 25 | 1 | 7 | 3 |
| e21.5 | TS 26 | 1 | 5 | 3 |
