# Supplementary figures and images for: A physiological and histological atlas of reproduction in the North American deer mouse (Peromyscus maniculatus)
Source: PLoS One. 2025 Jun 5;20(6):e0323266. doi: 10.1371/journal.pone.0323266 (PMC12140262; doi:10.1371/journal.pone.0323266)

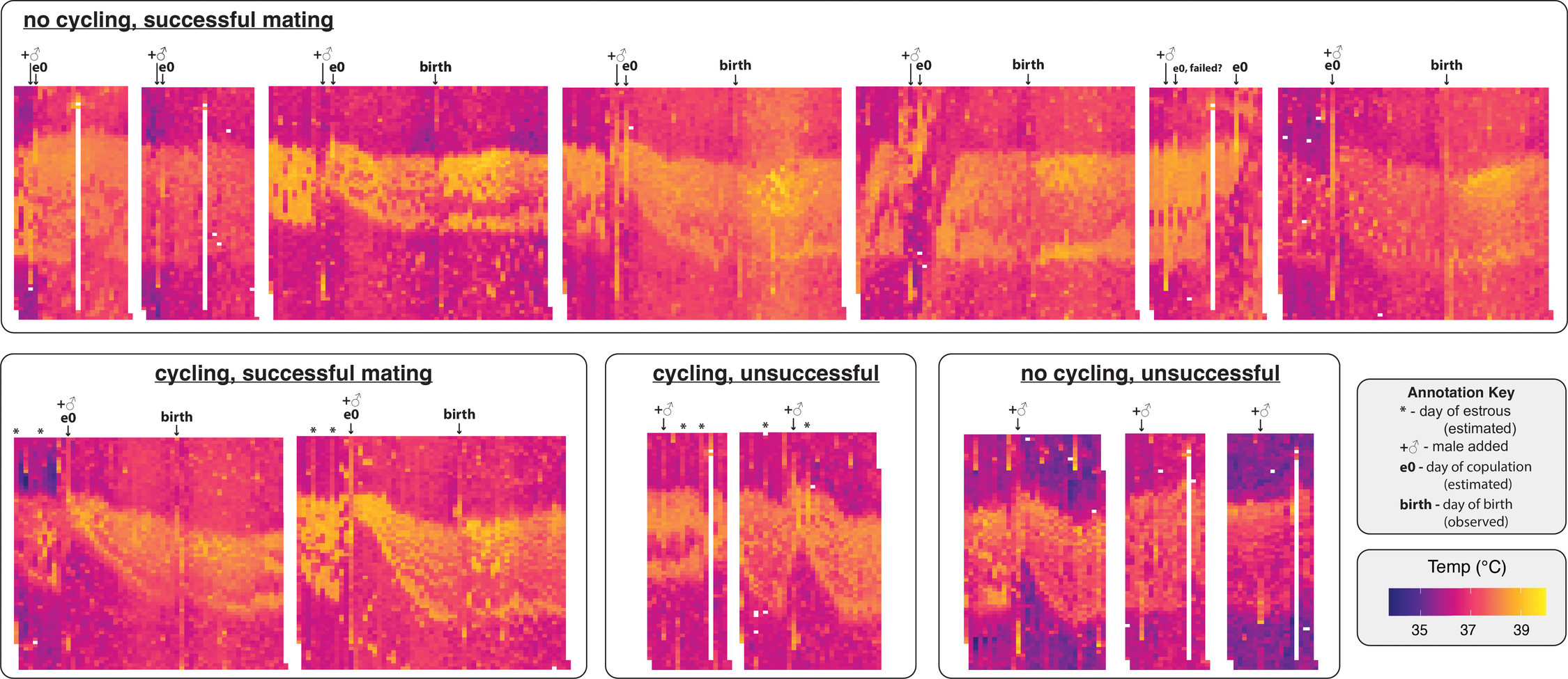

Supplement: S1 Fig — Warmer colors represent higher body temperatures and cooler colors represent lower body temperatures. Each column represents a single day, and each row is a single hour. Hours of the day run from 1200 (12:00 PM) at the bottom of the plot to 1159 (11:59 AM) at the top of the plot so that the active phase is centered. Individual temperature raster plots are grouped by type (cycling, not cycling) and outcome (successful matings that resulted in pregnancies or unsuccessful matings). Cyclicity was determined using qualitative assessments of fluctuations in rest-phase body temperature. Estimated days of estrous used to assign cyclicity is indicated using asterisks (*) in plot. All plots use an identical range of temperatures for color plotting (Temp Key in bottom right). White boxes in plots are missing data; we experienced one failure of the temperature reading system, which resulted in approximately 24 h of lost data in the middle of a subset of the experiments. (TIF) [file pone.0323266.s002.tif]

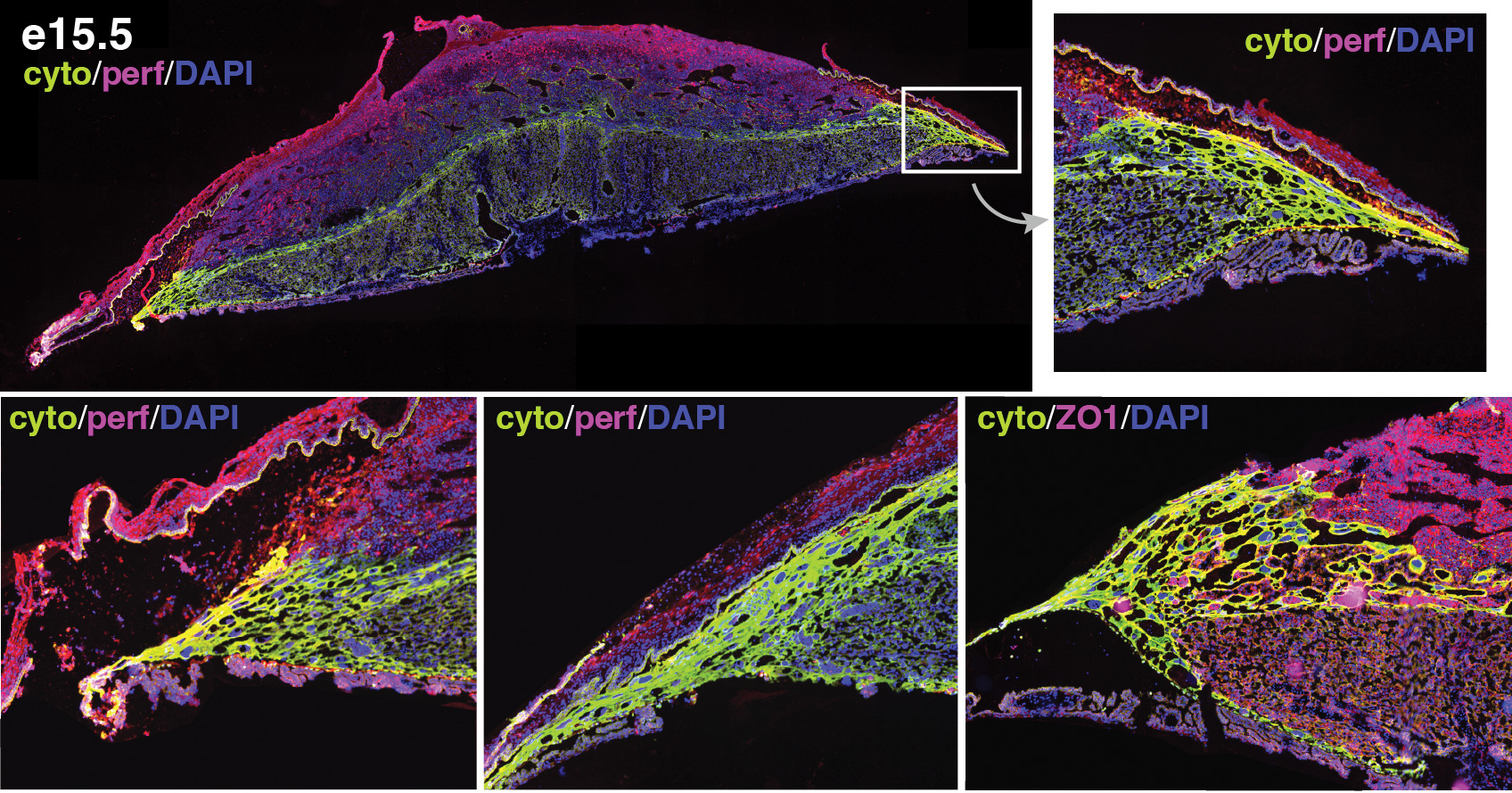

Supplement: S3 Fig — (Top) pTGCs accumulate at the edges of the placental disk in the junctional zone on embryonic day 15.5 (e15.5). The expanded image (right) shows further detail. (Bottom) Representative images of pTGC accumulation across sections show that the accumulation of pTGCs is common and extends to e16.5. These accumulations are largely ZO-1-negative (bottom right). (TIF) [file pone.0323266.s004.tif]
