# Supplementary material for: A physiological and histological atlas of reproduction in the North American deer mouse (Peromyscus maniculatus)
Source: PLoS One. 2025 Jun 5;20(6):e0323266. doi: 10.1371/journal.pone.0323266 (PMC12140262; doi:10.1371/journal.pone.0323266)
Supplement: Attachment — Submitted filename: Atlas_PLOSONE_revisions_Mar2025.docx [file pone.0323266.s006.docx]

Dear Dr. Wilsterman,

Your manuscript entitled "A physiological and histological atlas of reproduction in the North American deer mouse (Peromyscus maniculatus)” has been reviewed by experts in the subject.

The reviewers were of the opinion that the manuscript contains important information that is likely to be of interest to other investigators.

However, they also identified a number of comments that require further attention, as indicated in the reviews appended below.

I encourage you to respond to the reviewers' comments and satisfy the concerns raised in both critiques.

If you do decide to resubmit, it is important that you pay attention to all the major and minor issues raised in the reviews, since I will once again be asking the same reviewing editors for their opinion on any revised manuscript.

Please note that any invitation to submit a revised manuscript should not be taken as any guarantee of acceptance or publication, and that the decision to
resubmit rests solely with the authors.

Thank you for choosing PLOS ONE, and we look forward to your revised manuscript.
Kind regards,

Rupasri Ain
Academic Editor
PLOS ONE

We appreciate the editor and reviewer’s agreement that the submitted manuscript provides a unique and valuable resource for expanding our understanding of both Peromyscus biology specifically and, more broadly, comparative mammalian reproductive physiology. Both reviewers stated that the manuscript is technically sound, the data supports the conclusions, the statistical analysis was performed appropriately and rigorously, that underlying data were all made fully available, and that the manuscript was presented in intelligible English. We have not made any substantive changes to these aspects of the manuscript, except to clarify data and figure availability, as request by PLOS ONE (see below reviewer comments).

We have made revisions to the manuscript as suggested by the reviewers, and we provided specific responses to each comment below along with reference to the specific associated revisions.

**Reviewer #1:** Manuscript Number: PONE-D-25-03756
A physiological and histological atlas of reproduction in the North American deer
mouse (Peromyscus maniculatus)

Wilsterman et al effective establishes the importance of North American deer mouse (Peromyscus maniculatus) as a model organism in multiple biological fields particularly in reproductive biology that focused on characterizing body temperature profiles across reproductive stages and generating a detailed histological atlas of placental development. Results show that body temperature can be used to diagnose copulation and pregnancy in deer mice. Moreover, authors found a unique organization and behaviours of trophoblast cells, especially at the maternal-fetal interface in the deer mouse. The objectives of the study were clearly setup and contributed to a detailed comprehensive understanding of reproduction in deer mouse. The manuscript is nicely design and conceived and I indeed enjoyed reading the paper. I have some points for revision which I feel to compulsory address and incorporate in the manuscript (see my comments below and – for the ease of the authors). There are English grammatical mistakes and needs to be revised throughout the manuscript

We have worked to correct grammatical and clarity errors throughout, including those kindly pointed out by the reviewer.

As mentioned in the study that male presence disrupted female body temperature rhythms. It is unclear that male presence influenced the female reproductive physiology, for instance: did authors examine female cortisol levels for stress? and any changes in breeding behaviour of females?

We did not examine female corticosterone levels for stress, nor did we collect activity data that would have allowed us to assess breeding behaviors. We therefore are not able to speak to these queries by reviewer.

Moreover, on what basis author selected the gestational stages for placental analysis is not clearly justified. Are these stages were selected on earlier literature or studies??

We collected implantation sites spanning the period of placental development in deer mice, from the beginning of placental development (see main manuscript text and Fig. 3) through to the point at which placental development is stable, near term (see Fig. 4). Within that period, we collected implantation sites approximately very 24 h across development. These timepoints align with comparative work including [1,2]. During these experiments, we did miss collecting any animals at e19.5, however our data show that placental size and composition does not substantively change relative to e18.5 or e20.5, and thus we omitted to fil in this point.

Results show that cyclicity is not a reliable predictor of fertility in P. maniculatus, as only 2 cycling females became pregnant, and 7 were found to be non-cycling females. Did male presence, stress or other environmental factors influenced cyclicity detection?

We have no reason to suspect that stress, male presence, or any other specific environmental factors would have influenced cyclicity detection. All females were singly housed during this period in identical caging in a relatively quiet area within the vivarium. No animals showed any overt signs of stress (i.e., stereotypies). The body temperature data is all collected without animal disturbance (collected via a fixed plate PIT-tag reader). We have added further text to the materials and methods to clarify our approach:

“Animals were left nearly completely undisturbed during experimental pairing, gestation, and lactation outside of daily welfare checks and cage changes. The wild-derived strain used in this study tends to be highly sensitive to handling; we thus minimized disturbance or handling to avoid potential interference with reproductive parameters including body temperature.”

How frequently pseudo-pregnancy occurs in P. maniculatus.

We are not sure, and we don’t have the data to quantify the frequency of this. One female in our experiment (Fig. S1, first row, 6^th^ plot) did exhibit body temperature changes that could be considered consistent with pseudopregnancy or pregnancy loss. From these data, it certainly seems possible, but we do not have sufficient data to make more concrete statements about this point.

We expanded our text on this point in the main results section:

“We also observed a single incidence of potential pseudo-pregnancy (**S1** **Fig**, sixth panel from the left in the top row). In the instance of apparent pseudo-pregnancy, we observed an extended warm temperature signal similar to that observed on the estimated day of copulation for other females and a relatively warmer rest-phase body temperatures that was initially consistent with patterns we saw in females that became pregnant and later gave birth. However, unlike females that gave birth, the elevated rest phase temperature in this pseudo-pregnant female disappeared within a week. Furthermore, we then observed another extended warm phase aligned with estimated copulation associated with a subsequent successful pregnancy.”

Line 646. I suggest to move table 1 in results section from discussion

We have now moved this table to the results.

Line 598. Please change “in a other mammals to in other mammals”

Thank for this typo correction!

Line 668. Change to “specific”

From this comment, we could not infer exactly where the reviewer requested this insertion. Nonetheless, we did make minor revisions to the sentences in this section to increase specificity and clarity.

Line 611. Change to “are not clear to is not clear”

Thank you! We have corrected this error.

Line 654-655. Change to “Further physiological and histological studies are needed to clarify the roles of trophoblasts and uNK cells in vascular remodeling in deer mice”

Thank you for this suggestion to improve clarity. We have made this change.

**Reviewer #2:** Wilsterman et al have conducted have used remote temperature sensors to assay body temperature in wild caught Peromyscus maniculatus (deer mice) during mating, gestation, and lactation. In addition, they have conducted an immunohistological analysis of placental tissue. This research expands the model systems available for understanding mammalian implantation and gestation and highlights the similarities and differences between species. This work will be of interest to researchers working with Peromyscus and to the broader reproductive biology community.

My main criticism is that I would like to see more annotation on the figures that highlight the descriptive text. For instance, in supplemental figure 1 can you provide annotations that highlight the differences in the plots for cycling vs non-cycling females, or indicating the endometrial glands in figure 3 as referenced at line 354. Please apply this suggestion to all figures and not just these two examples.

We have worked to improve annotation across all figures without obscuring the images heavily. We have accordingly revised figure captions to align with these changes. We also removed some references to Fig 4 where additional annotation of the figure was not possible. Specific annotation additions to figures are noted below:

- We added additional annotations for Fig 1.
- We added annotation for endometrial glands and section orientation to Fig 3.
- We added arrowheads to Fig 4 to point to the ZO-1-positive cell clumps within the developing junctional zone.
- We added arrows to Fig 4 to the point to the appearance of maternal vasculature within the decidua that is lined by trophoblasts.
- We revised text reference to Fig 4 where additional annotation to the figure was not reasonable to incorporate.
- We added annotation for maternal (m) and fetal (f) blood spaces to Fig 7.
- We added an annotation key to S1 Fig and we added a marker for estrous (estimated). We also added clarity in the key about which annotations were observed versus estimated or speculated.

In the results section, can you state how copulation was determined? I believe you annotate copulation retrospectively using the temperature data and that you don’t have precise evidence. Or are you using the increase in temperature in the rest phase (daylight?) as evidence of copulation? I use a lab stock of P. maniculatus (BW) in my research and I do vaginal gavage to look for sperm as an indication of copulation. I came to Peromyscus research from Mus, so I was used to “mice” mating in the dark period. I have found that the BW deer mice will mate in the light period as I find sperm when I do checks in the morning and the afternoon. A female that does not have sperm present in the morning gavage can have sperm present in the afternoon gavage.

Copulation or conception (we cannot and do not distinguish between these events here) was estimated based on birth date or developmental stage of sampled dams. The increase in temperature in the rest phase aligns with this estimated date.

Although we know delayed implantation or development certainly occurs in deer mice, the data generated in this study suggests that the back-calculated day of conception consistently aligns with an increase in temperature. It is not clear whether that is a function of estrous or copulation, or both. However, as suggested by the reviewer, this elevated temperature indeed extends into the light phase substantively, even up to 6 h after lights-on. (see Fig 1 and Fig S1).

We did not use lavages to confirm copulation because our mice are generally highly sensitive to handling, especially relative to BWs (having worked with both). To avoid potential interference with reproductive parameters, we minimized disturbance or handling. Nonetheless, as indicated above, our body temperature data agree with your assessment that deer mice may be most likely to copulate early in the light phase.

We have worked to clarify these points across the materials and methods, main text, and figure captions. We have also added clarity to the figures to specify whether labeled events were estimated or observed.

In figure 2, please provide a code for your significance indicators.

The relevant information is now clarified in the Figure 2 caption:

“Different lowercase letters above each boxplot indicate statistically significant differences in daily maximum or minimum body temperature across stages (P < 0.05) in a linear mixed model that included dam ID as a random effect. Shared letters indicate no significant difference between groups. See Materials & Methods for further detail.”

At line 305, please indicate why you chose to start your analysis at e11.5.

As shown in Fig 3 and 4, e11.5 is the day or developmental stage immediately prior to placental development. Placental development emerges on e12.5 (see Fig 3). Because of our focus on placentation, we started at this timepoint.

We added the following text to clarify this point (see italics and underlined text):

“To describe normal placental development in deer mice, we generated a time course of placental development from e11.5 (the developmental stage at which the placenta begins to emerge) through near-term (**Fig 3 and 4**).”

In the figure 4 legend, please remove the reference to laminin data that isn’t shown.

Thank you – this text was indeed unnecessary.

When submitting your revision, we need you to address these additional requirements.

1. Please ensure that your manuscript meets PLOS ONE's style requirements, including those for file naming. The PLOS ONE style templates can be found at <https://journals.plos.org/plosone/s/file?id=wjVg/PLOSOne_formatting_sample_main_body.pdf> and <https://journals.plos.org/plosone/s/file?id=ba62/PLOSOne_formatting_sample_title_authors_affiliations.pdf>

We have reviewed these requirements and updated as was needed.

2. We note that you have indicated that there are restrictions to data sharing for this study. PLOS only allows data to be available upon request if there are legal or ethical restrictions on sharing data publicly. For more information on unacceptable data access restrictions, please see <http://journals.plos.org/plosone/s/data-availability#loc-unacceptable-data-access-restrictions>.

Before we proceed with your manuscript, please address the following prompts:

a) If there are ethical or legal restrictions on sharing a de-identified data set, please explain them in detail (e.g., data contain potentially identifying or sensitive patient information, data are owned by a third-party organization, etc.) and who has imposed them (e.g., a Research Ethics Committee or Institutional Review Board, etc.). Please also provide contact information for a data access committee, ethics committee, or other institutional body to which data requests may be sent.

b) If there are no restrictions, please upload the minimal anonymized data set necessary to replicate your study findings to a stable, public repository and provide us with the relevant URLs, DOIs, or accession numbers. For a list of recommended repositories, please see
<https://journals.plos.org/plosone/s/recommended-repositories>. You also have the option of uploading the data as Supporting Information files, but we would recommend depositing data directly to a data repository if possible.

We will update your Data Availability statement on your behalf to reflect the information you provide.

We have now made all raw image files available via Dryad at DOI: 10.5061/dryad.z34tmpgr7. All data, including data derived from images, and scripts are available via https://github.com/mjhemmerlein/Peromyscus-Gestational-Atlas

3. In the online submission form, you indicated that the data and scripts underlying the results presented in the study are available at <https://github.com/mjhemmerlein/Peromyscus-Gestational-Atlas>. Digital copies of all images shown or referenced in the paper can be obtained by contacting the corresponding author.

All PLOS journals now require all data underlying the findings described in their manuscript to be freely available to other researchers, either 1. In a public repository, 2. Within the manuscript itself, or 3. Uploaded as supplementary information.
This policy applies to all data except where public deposition would breach compliance with the protocol approved by your research ethics board. If your data cannot be made publicly available for ethical or legal reasons (e.g., public availability would compromise patient privacy), please explain your reasons on resubmission and your exemption request will be escalated for approval.

See above. All relevant data and images are now available.

4. Please amend the manuscript submission data (via Edit Submission) to include author Kathryn Wilsterman.

This has been corrected.

5. Please amend your authorship list in your manuscript file to include author Kate Wilsterman.

This has been corrected.

6. [Please remove all personal information, ensure that the data shared are in accordance with participant consent, and re-upload a fully anonymized data set.

Note: spreadsheet columns with personal information must be removed and not hidden as all hidden columns will appear in the published file.

Additional guidance on preparing raw data for publication can be found in our Data Policy (https://journals.plos.org/plosone/s/data-availability#loc-human-research-participant-data-and-other-sensitive-data) and in the following article: http://www.bmj.com/content/340/bmj.c181.long.](http://track.editorialmanager.com/CL0/http:%2F%2Fwww.bmj.com%2Fcontent%2F340%2Fbmj.c181.long./1/010f019569c6e887-f50fba6b-5567-4c9c-a03f-3532c835d4a4-000000/6dHXRzTiB-8zZLWbqqRduNlJFyhcX7flPh9nCBwlAnQ=201)

This has been implemented.

7. Please include captions for your Supporting Information files at the end of your manuscript, and update any in-text citations to match accordingly. Please see our Supporting Information guidelines for more information: <http://journals.plos.org/plosone/s/supporting-information>.

Captions for Supporting Information files are included at the end of the manuscript.

8. Please review your reference list to ensure that it is complete and correct. If you have cited papers that have been retracted, please include the rationale for doing so in the manuscript text, or remove these references and replace them with relevant current references. Any changes to the reference list should be mentioned in the rebuttal letter that accompanies your revised manuscript. If you need to cite a retracted article, indicate the article’s retracted status in the References list and also include a citation and full reference for the retraction notice.

We have checked this information. None of the cited publications have been retracted to our knowledge.
